# The effect of adding goal-directed hemodynamic management for elective patients in an established enhanced recovery program for colorectal surgery: results of quasi-experimental pragmatic trial

**DOI:** 10.1186/s13741-020-00163-3

**Published:** 2020-11-23

**Authors:** Matthew D. McEvoy, Jonathan P. Wanderer, Yaping Shi, Krishnan S. Ramanujan, Timothy M. Geiger, Matthew S. Shotwell, Andrew D. Shaw, Alexander T. Hawkins, Barbara J. Martin, Michael G. Mythen, Warren S. Sandberg

**Affiliations:** 1 Department of Anesthesiology, Vanderbilt University Medical Center, 1301 Medical Center Drive, TVC 4648, Nashville, TN 37232 USA; 2 Department of Biostatistics, Vanderbilt University Medical Center, 1301 Medical Center Drive, TVC 4648, Nashville, TN 37232 USA; 3 Department of Surgery, Vanderbilt University Medical Center, 1301 Medical Center Drive, TVC 4648, Nashville, TN 37232 USA; 4 Department of Anesthesiology & Pain Medicine, University of Alberta, Edmonton, Alberta Canada; 5 Department of Quality, Safety & Risk Prevention, Vanderbilt University Medical Center, 1301 Medical Center Drive, TVC 4648, Nashville, TN 37232 USA; 6 University College London Hospitals National Institute of Health Research Biomedical Research Centre, London, UK

**Keywords:** Enhanced recovery program, Goal-directed, Fluid, Hemodynamics, Colorectal surgery, Monitor, Cardiac output, Blood pressure, Outcomes

## Abstract

**Background:**

Recent literature has demonstrated that hemodynamic instability in the intraoperative period places patients at risk of poor outcomes. Furthermore, recent studies have reported that stroke volume optimization and protocolized hemodynamic management may improve perioperative outcomes, especially surgical site infection (SSI), in certain high-risk populations. However, the optimal strategy for intraoperative management of all elective patients within an enhanced recovery program remains to be elucidated.

**Methods:**

We performed a pre-post quasi-experimental study to assess the effect of adding goal-directed hemodynamic therapy to an enhanced recovery program (ERP) for colorectal surgery on SSI and other outcomes. Three groups were compared: “Pre-ERP,” defined as historical control (before enhanced recovery program); “ERP,” defined as enhanced recovery program using zero fluid balance; and “ERP+GDHT,” defined as enhanced recovery program plus goal-directed hemodynamic therapy. Outcomes were obtained through our National Surgical Quality Improvement Program participation.

**Results:**

A total of 623 patients were included in the final analysis (Pre-ERP = 246, ERP = 140, and ERP + GDHT = 237). Demographics and baseline clinical characteristics were balanced between groups. We did not observe statistically significant differences in SSI or composite complication rates in unadjusted or adjusted analysis. There was no evidence of association between study group and 30-day readmission. American Society of Anesthesiologists status ≥ 3 and open surgical approach were significantly associated with increased risk of SSI, composite complication, and 30-day readmission (*p* < 0.05 for all) in all groups.

**Conclusions:**

There was no evidence that addition of goal-directed hemodynamic therapy for all patients in an enhanced recovery program for colorectal surgery affects the risk of SSI, composite complications, or 30-day readmission. Further research is needed to investigate whether there is benefit of goal-directed hemodynamic therapy for select high-risk populations.

**Trial registration:**

NCT03189550. Registered 16 June 2017–Retrospectively registered, https://www.clinicaltrials.gov/ct2/results?cond=&term=NCT03189550&cntry=&state=&city=&dist=

## Background

Improving outcomes for surgical patients through goal-directed hemodynamic therapy (GDHT) and enhanced recovery programs (ERPs) has been the focus of major research initiatives for the past 25 years. ERPs have been shown to improve patient outcomes across many types of surgery, with the largest body of evidence surrounding colorectal surgery (Page et al., 2015; Thiele et al., 2015; Zhuang et al., 2015; Wang et al., 2015; Simpson et al., 2015). Of the many components listed for ERPs for colorectal surgery, GDHT and monitoring of cardiac output are given a ‘strong’ recommendation (Gustafsson et al., 2013; Nygren et al., 2013). However, recent meta-analyses of GDHT trials have yielded mixed results, with one reporting improved outcomes, three reporting a decrease in morbidity but not mortality, and one reporting a decrease in mortality but not morbidity (Feng et al., 2018; Sun et al., 2017; Ripolles-Melchor et al., 2016; Som et al., 2017). Recent trials investigating the effects of goal-directed fluid or hemodynamic therapy showed no benefit (Pestana et al., 2014). On the other hand, three recent large clinical trials have reported positive effects from GDHT for both low to moderate and high-risk surgical patients (Calvo-Vecino et al., 2018; Pearse et al., 2014; Futier et al., 2017).

It is unclear how the results of GDHT trials generalize in the setting of ERPs (Bloomstone & Dull, 2018). None of the studies included in the meta-analyses of GDHT were in the setting of an ERP (Feng et al., 2018; Sun et al., 2017; Ripolles-Melchor et al., 2016; Som et al., 2017), which is also true for the recent GDHT trials (Calvo-Vecino et al., 2018; Pearse et al., 2014; Futier et al., 2017). Prior research has reported that stroke volume or cardiac output optimization and protocolized intraoperative hemodynamic management may improve outcomes in certain high-risk populations (Cecconi et al., 2013; Arulkumaran et al., 2014). However, the optimal strategy for intraoperative management of patients within an ERP remains to be elucidated (Bloomstone & Dull, 2018; Gupta & Gan, 2016). We hypothesized that the addition of continuous, non-invasive cardiac output monitoring with protocolized hemodynamic management for all patients undergoing colorectal surgery within an established ERP would result in decreased rates of surgical site infection (SSI) and improvement in other postoperative outcomes (McEvoy et al., 2016).

## Methods

We performed a pre-post quasi-experimental pragmatic trial where all included patients received the standard of care at the time of their surgical intervention. This study was approved by the Institutional Review Board (IRB) (protocol #140558) at our institution (Vanderbilt University Medical Center, Nashville, TN, USA), with waiver of requirement for written informed patient consent, as all care components were standard of care. The registration number for the study is NCT03189550 on ClinicalTrials.gov (http://www.clinicaltrials.gov). Matthew D. McEvoy, MD, is the principal investigator; the date of registration is 16 June 2017, which was prior to obtaining data for analysis.

We have previously published the results of our ERP that included the concept of targeting zero fluid balance but did not include algorithm-driven GDHT with continuous non-invasive cardiac output monitoring (McEvoy et al., 2016). Based upon research demonstrating improved outcomes from GDHT with pulse contour analysis, a large-scale quality improvement project was undertaken to advance the ERP for colorectal surgery at our institution through the addition of an intraoperative GDHT management algorithm that included continuous, non-invasive cardiac output monitoring for all patients undergoing colorectal surgery. This manuscript adheres to the applicable Standards for Quality Improvement Reporting Excellence (SQUIRE).

Two hundred fifty consecutive patients undergoing elective colorectal surgery including resection of bowel with or without ostomy creation and receiving care within our ERP between 3 March 2015 and 17 February 2017 were included in the study (ERP + GDHT). All care components delivered to these patients were standard of care. A priori, an allocation and analysis plan was created to compare these patients with an historical control group consisting of 250 elective colorectal surgery patients having bowel resection with or without ostomy creation cared for between 9 February 2013 and 26 June 2014, prior to implementation of the colorectal ERP (Pre-ERP), as well as all 146 elective colorectal surgery patients having bowel resection with or without ostomy creation cared for between 27 June 2014 and 2 March 2015, between the inception of the ERP and the start of this quality improvement initiative (ERP). All patients in the groups were included consecutively during their time period. Specifically, the patients in the ERP + GDHT group were enrolled consecutively as noted above. All elective colorectal surgery patients were included from the ERP period. The patients from the pre-ERP group were added to the group by going backwards consecutively from the ERP launch date until 250 patients were included. Emergency cases were excluded, including any case with a preoperative bowel perforation.

Based on prior studies showing a difference in the rate of SSI with GDHT, the primary outcome was the rate of SSI, with secondary outcomes including length of stay, hospital readmission, and a composite complication rate composed of NSQIP (National Surgical Quality Improvement Program)—defined outcomes grouped as respiratory, transfusion, acute kidney injury, urinary tract infection, sepsis, cardiac, and hematologic.

**Fig. 1:**
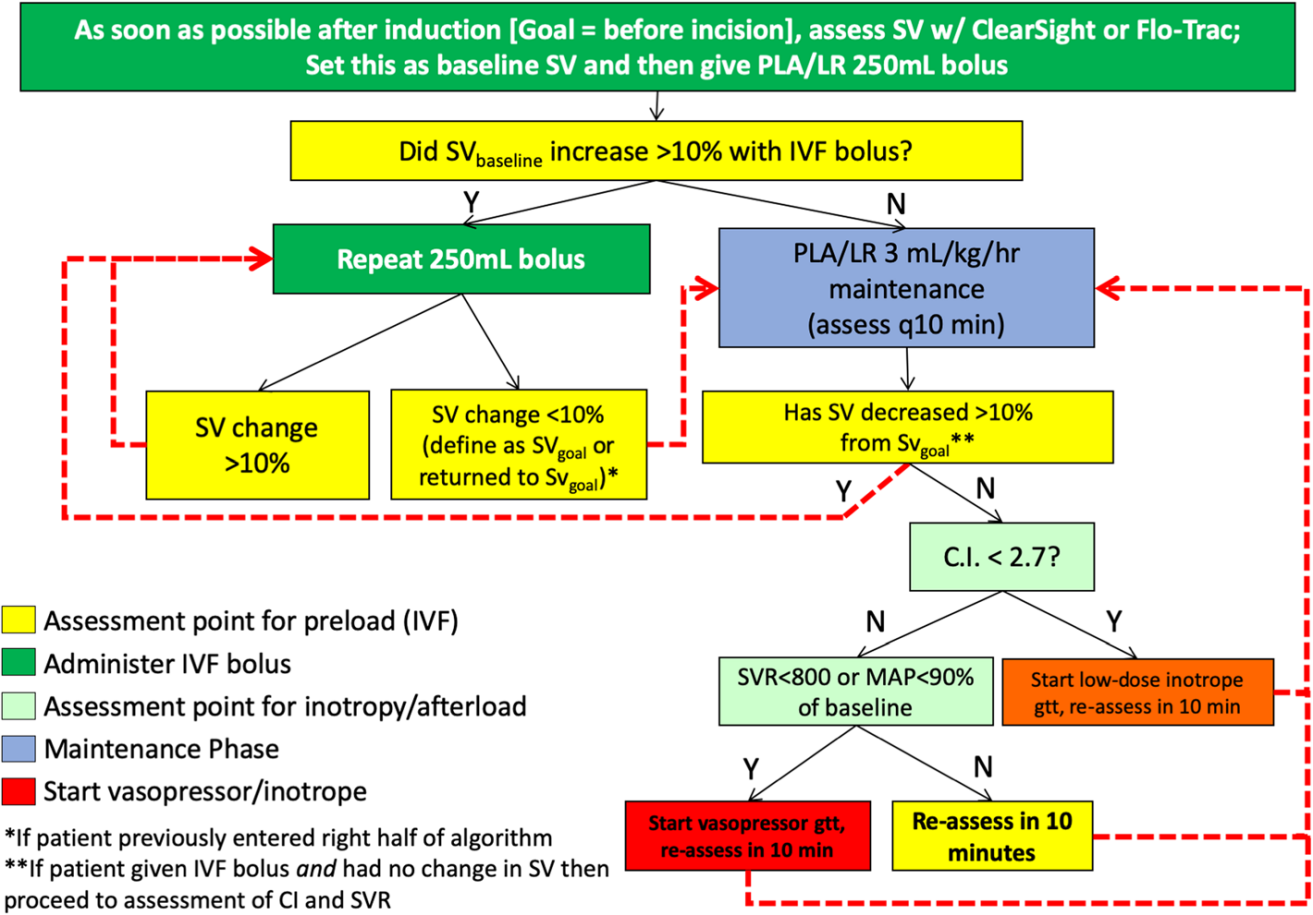
Goal-directed hemodynamic therapy algorithm. This figure illustrates the goal-directed hemodynamic therapy algorithm that was employed. Stroke volume was monitored with ClearSight sensor and EV1000 clinical platform (Edwards Lifesciences, Irvine, CA). SV: stroke volume; PLA: Plasma-Lyte A; LR: lactated Ringer’s; IVF: intravenous fluid; mL: milliliter; kg: kilogram; hr: hour; CI: cardiac index; SVR: systemic vascular resistance; MAP: mean arterial pressure; gtt: infusion

For the patients receiving GDHT, a management algorithm was followed (see Fig. 1). This management protocol was made available to the department as part of the ERP care pathways and was reviewed with all in-room providers (resident or nurse anesthetist) and the assigned anesthesiologist prior to each case. A research assistant was present for monitor calibration and was immediately available for any question concerning the monitor or the protocol. If the anesthesiologist had any questions that involved any clinical decision-making, these were referred to the principal investigator (MDM).

All patient data were collected prospectively through routine clinical care and standardized NSQIP abstracting and stored in the perioperative data warehouse (PDW), an IRB approved data repository (protocol #120365; waiver of consent approved under that study protocol). Hemodynamic data from the patients in the ERP+GDHT group were collected prospectively from the monitoring device (ClearSight sensor and EV1000 clinical platform, Edwards Lifesciences, Irvine, CA, USA) and stored in REDCap after each case (Harris et al., 2009). Patient outcome data were collected by trained abstractors according to the American College of Surgeons (ACS) NSQIP guidelines. Our institution participates in the ACS NSQIP Procedure Targeted program, which includes reporting for all colorectal procedures. Published NSQIP definitions for all the demographic and outcome data were strictly followed (Program ACoSNSQI, n.d.). The abstractors do not deliver clinical care in the operating room and were not aware of the ERP + GDHT implementation until after the study was completed. The Case Mix Index (CMI) of each population is reported as well. CMI is used in USA healthcare to give weight to certain medical conditions around the perioperative period in order to quantify the comorbid status of patient populations. The CMI of a patient population reflects the diversity, clinical complexity, and resource needs of all the patients in that population. A higher CMI indicates a more complex and resource-intensive case load.

### Statistical analysis

Patient demographics and clinical characteristics were summarized using the median (25th and 75th percentile) for continuous variables and percentages for categorical variables. The Kruskal-Wallis test and the Pearson chi-square test were performed as appropriate to compare differences among three study groups (i.e., PreERP, ERP, ERP + GDHT). Generalized linear mixed effects regression was used to examine the association between study group and the binary outcomes (SSI, readmission in 30 days) while adjusting for a set of pre-specified potential confounders that included age, body mass index (BMI), sex, American Society of Anesthesiologists (ASA) physical status classification, and surgical approach as fixed effects, and surgical procedure (current procedural terminology [CPT] code) as a random effect. The secular trends over time introduced by unobserved time-dependent confounders were investigated using a segmented regression approach, specifically by adding a time-by-study group interaction in the model. Similarly, linear mixed effects regression was fit for log transformed hospital length of stay (LOS) adjusting for the same set of covariates. Effect estimates were presented using the odds ratio and ratio of medians (OR/RM, 95% CI, *P* value).

Finally, in order to evaluate adherence to the GDHT management algorithm, a time-in-target analysis was undertaken to evaluate the time for which the patient had 1, 2, or 3 of the hemodynamic variables in the target range. The hemodynamic algorithm goals evaluated were cardiac index (CI) > 2.7 L/min/m^2^, mean arterial pressure (MAP) > 65 mmHg, and systemic vascular resistance (SVR) > 800 dynes*s/cm^5^. These variables were based on a compilation of prior studies in GDHT (Feng et al., 2018; Sun et al., 2017).

For a pairwise comparison of study groups, having 250 participants in each group provides approximately 80% power to detect an odds ratio of 0.45 and 0.20 or smaller with regard to the rate of SSI, assuming that the pre-ERP incidence is 18% and 7%, respectively. Thus, the current study was sufficiently powered to detect a large, clinically meaningful effect of ERP+GDHT on the incidence of SSI in this population. In addition, having 250 participants in each group provides approximately 95% power to detect a 1-day difference in average LOS, assuming a pre-ERP mean of 5 (SD 3) days.

All analyses were performed using the R Programming Language 3.3.0 (R Foundation for Statistical Computing, Vienna, Austria). We tested for statistical significance at 0.05 significance level.

## Results

**Fig. 2:**
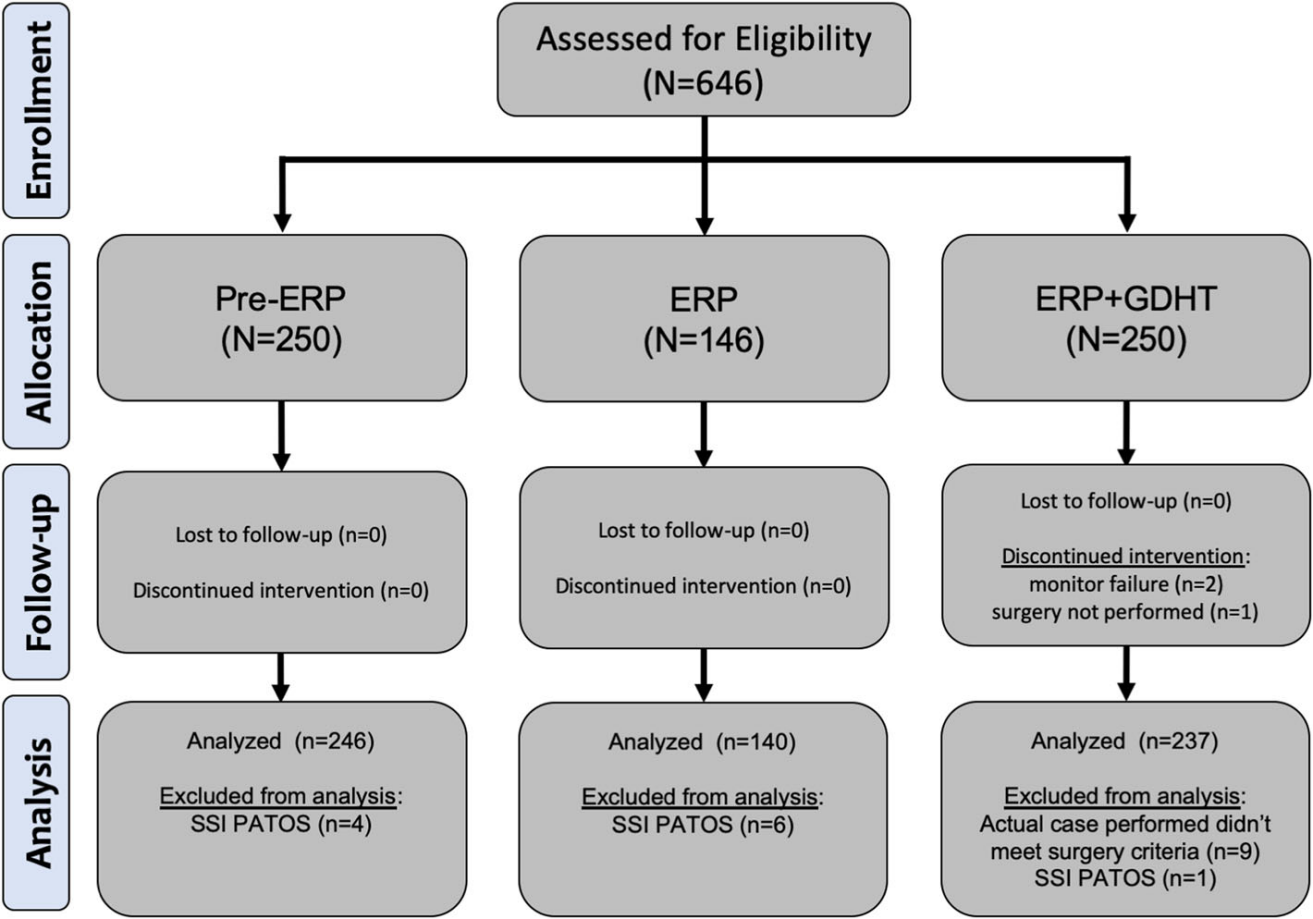
CONSORT flow diagram. CONSORT flow diagram illustrating patient enrollment, allocation, follow-up, and analysis. ERP: enhanced recovery program; GDHT: goal-directed hemodynamic therapy; PATOS: present at time of surgery; CONSORT: consolidated standards of reporting trials

A total of 646 patients were assessed for eligibility. Nine patients in the ERP + GDHT group did not meet inclusion criteria based upon the actual surgery performed; two patients were excluded as the non-invasive monitor did not function properly (e.g., routinely displayed error messages or no hemodynamic variables during the case); one patient had the case aborted after induction of anesthesia and before incision owing to the development of unstable atrial fibrillation. After removing 11 patients because of SSI being present at time of surgery (PATOS) (Pre-ERP = 4, ERP = 6, and ERP + GDHT = 1), the final data set included 623 patients for analysis (Fig. 2).

### Patient demographics and case characteristics

Patient demographics were reasonably balanced between study groups (Table 1). A complete listing of intraoperative variables for all groups can be found in Table 2. Of note, median surgery and anesthesia times were longer and median inspired oxygen and end-tidal carbon dioxide concentrations were higher in the ERP and ERP+GDHT groups versus Pre-ERP (*P* < 0.01). Total intravenous fluids were lower in the ERP and ERP + GDHT as was use of colloids (*P* < 0.01). Use of vasopressor and inotrope boluses as well as the use of vasopressor infusions increased in the ERP+GDHT periods (*P* ≤ 0.01). However, there was no increase in the use of inotrope infusions.

**Table 1:** Demographics and baseline characteristics

Characteristic	Pre-ERP (***n*** = 246)	ERP (***n*** = 140)	ERP + GDHT (***n*** = 237)	***P*** value
**Age**	58 (42, 67)	53 (39, 66)	56 (38, 66)	0.50
**Female [*N*** **(%)]**	132 (53.7%)	72 (51.4%)	105 (44.5%)	0.072
**BMI**	26 (23, 30)	27 (24, 31)	27 (23, 32)	0.43
**ASA physical status class [*N*, (%)]**				0.64
I/II	110 (44.7%)	67 (47.9%)	101 (42.8%)	
III/IV	136 (55.3%)	73 (52.1%)	136 (57.2%)	
**Case mix index**	1.75 (1.64, 2.57)	2.55 (1.66, 2.56)	2.55 (1.56, 2.55)	0.17
**Functional health status**^**a**^ **[*N*, (%)]**				0.22
Independent	243 (98.8%)	139 (99.3%)	234 (98.6%)	
Partially dependent	3 (1.2%)	0 (0%)	3 (1.4%)	
Totally dependent	0 (0%)	1 (0.7%)	0 (0%)	
**Pre-existing conditions [*N*, (%)]**				
Diabetes	21 (8.5%)	12 (8.6%)	21 (8.9%)	0.98
Current smoker within 1 year	46 (18.7%)	27 (19.3%)	33 (14.0%)	0.27
Severe COPD	11 (4.5%)	2 (1.4%)	6 (2.5%)	0.23
Dialysis	0 (0%)	2 (1.4%)	2 (0.8%)	0.12
Disseminated cancer	7 (2.8%)	5 (3.6%)	3 (1.3%)	0.35
Open wounds	4 (1.6%)	3 (2.1%)	1 (0.4%)	0.35
On steroid/immunosuppressant	76 (30.9%)	51 (36.4%)	83 (35.2%)	0.48
> 10% weight loss in 6 months	14 (5.7%)	10 (7.1%)	15 (6.4%)	0.86
Transfusion 72 h prior to surgery	0 (0%)	0 (0%)	2 (0.8%)	0.19

^a^Functional health status is only available for 211 patients in the ERP+GDHT cohort. Continuous variables are shown in median (25th, 75th percentile); categorical data are shown in number and (percentage (%)).

*ERP* enhanced recovery program, *GDHT* goal-directed hemodynamic therapy, *n* number, % percentage; *BMI* body mass index, *ASA* American Society of Anesthesiologists, *COPD* chronic obstructive pulmonary disease, *SIRS* systemic inflammatory response syndrome

**Table 2:** Intraoperative variables

Variable	Pre-ERP (***n*** = 246)	ERP (***n*** = 140)	ERP + GDHT (***n*** = 237)	***P*** value
**Times**				
Surgery time (min)	144 (108, 195)	178 (135, 237)	165 (122, 214)	< 0.01
Anesthesia time (min)	179 (144, 237)	222 (179, 282)	206 (164, 263)	< 0.01
**Use of epidural analgesia,** ***N*** **(%) of patients receiving**	16 (6.5%)	16 (11.4%)	11 (4.7%)	0.04
**Surgical site infection prevention**				
Pre-incision antibiotics %	246 (100%)	140 (100%)	236 (99.6%)	0.44
Median temperature, °C	36.4 (36.1, 36.7)	36.3 (36.0, 36.7)	36.0 (35.7, 36.3)	< 0.01
Fraction of inspired oxygen	0.60 (0.55, 0.80)	0.81 (0.76, 0.84)	0.75 (0.59, 0.80)	< 0.01
End-tidal carbon dioxide, mmHg	34 (32, 38)	38 (35, 39)	37 (34, 39)	< 0.01
**Glucose**				
Preoperative, mg/dL	95 (87,106)	102 (92,123)	105 (92,124)	< 0.01
Recovery room, mg/dL	146 (127,168)	144 (122,172)	158 (138,178)	0.11
Recovery room glucose > 180, *N* (%) of patients	9 (3.7%)	17 (12.1%)	17 (7.2%)	0.01
**Intraoperative intravenous fluid use**				
Total Intravenous fluids, mL	2125 (1700,2925)	1900 (1450,2500)	1800 (1500,2300)	< 0.01
Crystalloid, mL	2000 (1600,2800)	1800 (1450,2428)	1800 (1500,2300)	< 0.01
Plasmalyte-A or lactated r inger’s, mL	2000 (1500,2600)	1700 (1400,2400)	1800 (1438,2200)	0.04
Colloid, *N* (%)	36 (14.6%)	9 (6.4%)	15 (6.4%)	< 0.01
Albumin, *N* (%)	35 (14.2%)	9 (6.4%)	14 (5.9%)	< 0.01
Fresh frozen plasma, *N* (%)	0 (0%)	0 (0%)	0 (0%)	0.80
Packed red blood cells, *N* (%)	4 (1.6%)	0 (0%)	0 (0.8%)	0.43
Blood loss, mL	50 (50,100)	75 (29,150)	73 (30,150)	0.98
**Vasopressor drug use**				
Vasopressor bolus, *N* (%)	142 (57.7%)	97 (69.3%)	169 (71.2%)	< 0.01
Vasopressor infusion, *N* (%)	9 (3.7%)	14 (10.0%)	53 (22.5%)	< 0.01
Inotrope bolus, *N* (%)	103 (41.9%)	50 (35.7%)	122 (51.3%)	0.01
Inotrope infusion, *N* (%)	0 (0%)	1 (0.7%)	2 (0.8%)	0.37
**Disposition from operating room (% going to each destination)**				
Surgical ward, %	246 (100%)	139 (99.3%)	236 (99.6%)	0.46
Intensive care unit, %	0 (0%)	1 (0.7%)	1 (0.4%)	

*ERP* enhanced recovery program, *GDHT* goal-directed hemodynamic therapy, median temperature denotes the average of the median temperature of patients in each group, *vasopressor* phenylephrine, norepinephrine, or vasopressin, *intotrope* ephedrine, epinephrine, dobutamine, or dopamine. Continuous variables are shown in median (25th, 75th percentile), categorical data are shown in percentage (%). *P* values were calculated using the Wilcoxon rank sum test for continuous variables and the Kruskal-Wallis test for categorical variables. *P* values for any differences among groups

Surgical case characteristics are shown in Table 3. There were more laparoscopic surgeries in the Pre-ERP group (76.4%) as compared to ERP (67.9%) and ERP + GDHT (65.1%) groups (*P* = 0.023). There was also a difference in the percentage of proctectomy cases in the Pre-ERP group (12.2%) as compared to ERP (27.9%) and ERP + GDHT (20.8%) groups (*P* < 0.001).

**Table 3:** Case characteristics

Variable	Pre-ERP (***n*** = 246)	ERP (***n*** = 140)	ERP + GDHT (***n*** = 237)	***P*** value
**Surgery type**				< 0.001
Colectomy	216 (87.8%)	101 (72.1%)	188 (79.2%)	
Proctocolectomy	30 (12.2%)	39 (27.9%)	49 (20.8%)	
**Laparoscopic cases**	188 (76.4%)	95 (67.9%)	154 (65.1%)	0.023
**Wound classification**				0.085
Clean	1 (0.4%)	0 (0%)	0 (0%)	
Clean contaminated	157 (63.8%)	70 (50.0%)	138 (58.1%)	
Contaminated	58 (23.6%)	46 (32.9%)	62 (26.3%)	
Dirty/infected	30 (12.2%)	24 (17.1%)	37 (15.7%)	
**Surgical wound closure**				0.14
All layers closed	244 (99.2%)	139 (99.3%)	230 (97.0%)	

Data are shown as the number and percentage (%) of patients per group.

*ERP* enhanced recovery program, *GDHT* goal-directed hemodynamic therapy, *n* number

### Outcomes

**Fig. 3:**
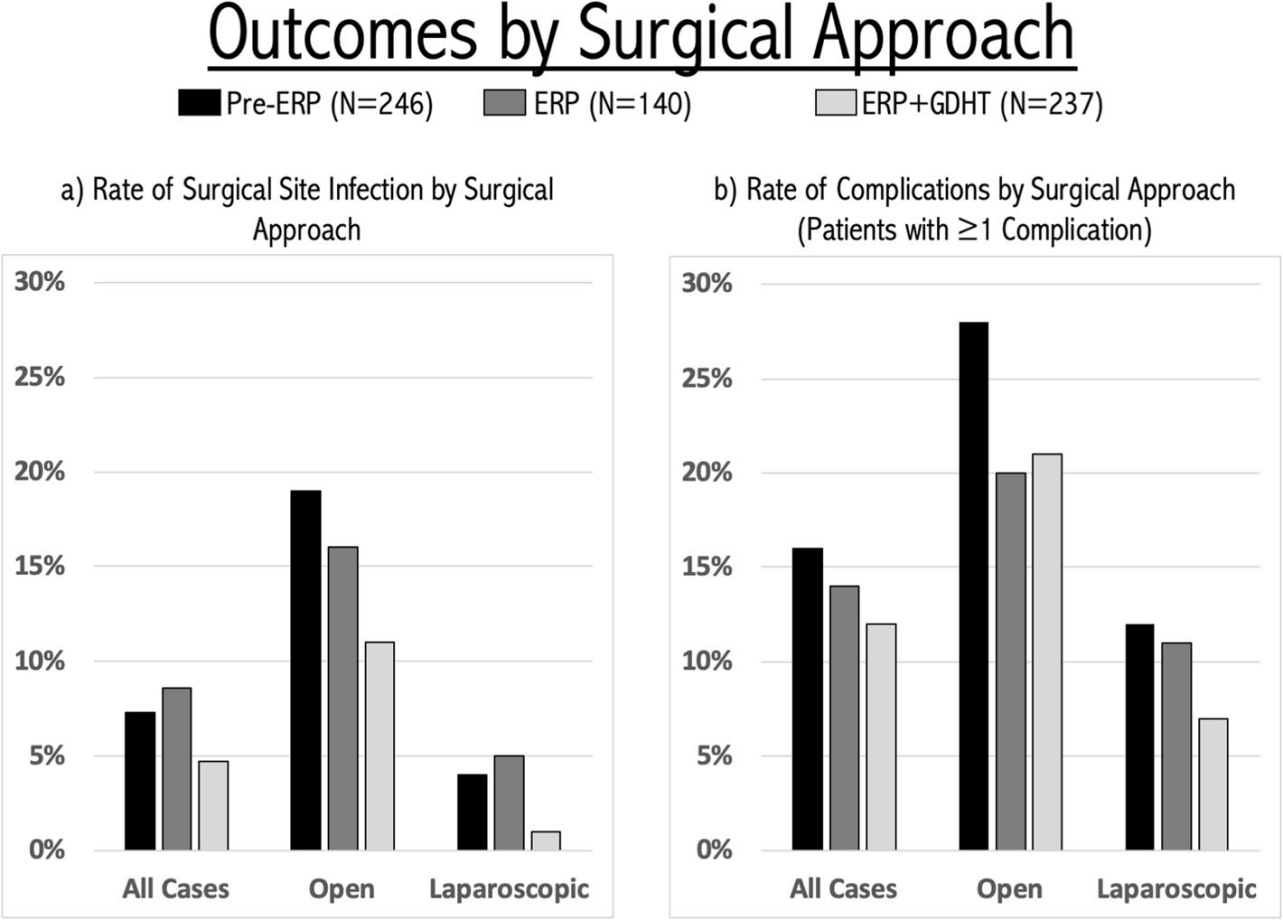
**a**, **b** Percentage of patients with surgical site infection and any complication by surgical approach. This figure illustrates the percentage of patients with a surgical site infection (**a**) and overall complications (**b**) by surgical approach by study phase. There were no statistically significant differences between groups in any phase. ERP: enhanced recovery program; GDHT: goal-directed hemodynamic therapy

There was no evidence of a significant difference in SSI rate or composite complication rate among the three groups before or after adjustment for covariates, although the absolute rates of both outcomes were smaller in ERP + GDHT versus Pre-ERP or ERP samples (Fig. 3a, b). Significantly higher SSI rate was observed for open compared to laparoscopic surgeries overall (15% vs. 3%, *P* < 0.001), in Pre-ERP (19% vs. 4%, *P* < 0.001), in ERP (16% vs. 5%, *P* = 0.04), and in ERP+GDHT (11% vs. 1%, *P* < 0.001). In the adjusted analysis, we examined whether GDHT was associated with reduced SSI rates for either laparoscopic or open surgery patients by testing the interaction of study group by surgery approach. Although the observed rate of SSI was smaller, there was insufficient statistical evidence of a difference for either approach. Due to the rarity of respiratory, transfusion, acute kidney injury, urinary tract infection, sepsis, cardiac, and hematologic complications, as well as readmission within 7 days of surgery, only descriptive statistics are presented (see Additional file 1, Table 1).

The median hospital length of stay (LOS) was 4.2, 3.4, and 4.0 days in Pre-ERP, ERP, and ERP + GDHT groups, respectively (*P* = 0.10). The reduction in LOS from Pre-ERP to ERP was marginal. In unadjusted comparisons, no significant difference in LOS was observed between ERP and Pre-ERP (*P* = 0.33) or between ERP and ERP+GDHT (*P* = 0.71). However, after adjusting for covariates the median LOS reduced by 12.9% (95% CI 0.01–23.8, *P* = 0.04) in ERP compared to Pre-ERP. There was no association between ERP + GDHT and readmission in 30 days in the adjusted analysis.

ASA status ≥ 3 was associated with increased risk of SSI (*P* = 0.003), any complication (*P* < 0.001), and readmission within 30 days (*P* = 0.027). Open (as opposed to laparoscopic) surgical approach was associated with SSI (*P* < 0.001), any complications (*P* = 0.003), and hospital LOS (*P* < 0.001). There was no significant trend over time for any outcome.

### Analysis of goal-directed therapy compliance

Additional file 1, Table 2 (“Time in Target Analysis”) shows the complete results of this analysis. Overall, patients met mean arterial pressure (MAP), systemic vascular resistance (SVR), and cardiac index (CI) goals 94.1%, 86.6%, and 85.9% of the time, respectively. Two out of 3 of the target parameters were maintained 94.7% of the time. However, all 3 of the parameters were only achieved 36.1% of the time. There was no association between time-in-target and patient outcomes.

## Discussion

We performed a pragmatic pre-post study to assess the effects of applying continuous cardiac output monitoring and individualized GDHT for all patients undergoing colorectal surgery within an established ERP and found no significant difference in outcomes. There may be several reasons why there was no evidence in this study of a differential benefit for adding GDHT within our ERP. First, SSI (primary outcome) was already at a low rate after the introduction of ERP without GDHT. In fact, our institutional SSI and overall complication rate is within the top performers among NSQIP institutions, which may make further improvement challenging (Hawkins et al., 2019). Additionally, the LOS in patients undergoing colorectal surgery at our institution was lower in all three groups than that reported in any intervention group from the recent large trials in which GDHT was found to be of benefit (Calvo-Vecino et al., 2018; Pearse et al., 2014; Futier et al., 2017). Thus, the ability to show additive benefit of GDHT on complications or LOS for all patients within an established ERP may be more difficult (Bloomstone & Dull, 2018; Gupta & Gan, 2016).

It is worthwhile to note that if a GDHT protocol is intended to achieve a high level of compliance with all three hemodynamic targets (i.e., a hemodynamic target zone of normal MAP, afterload, and cardiac output), then the average “dose” in our study is low (i.e., all 3 variables simultaneously in target range < 40% of time). Our finding of achieving the goal of MAP > 65 mmHg approximately 95% of the time accords with previous reports (Maheshwari et al., 2018). However, this needs to be further researched, as we found no difference in outcomes even when 2 out of 3 variables were in target at almost all times (95%) in the ERP + GDHT group. As this was a pragmatic trial that reflects real-world practice, these results should be factored into any subsequent trial design. Perhaps greater computation/automation with closed loop systems administering fluids and medications could achieve a very high level of compliance by not only reacting to changes, but possibly predicting them based on recent hemodynamic patterns (Joosten et al., 2019). Consistent with proposed guidelines (Thiele et al., 2016), our data suggest that GDHT for low risk patients in established ERPs is very unlikely to be cost effective and larger trials are not justified.

However, for higher risk patients, and in particular open surgeries, major complications still occur despite implementation of enhanced recovery principles. In fact, we did find that high-risk patients (e.g., ASA ≥ 3, open approach) had much higher rates of SSI and all complications than those patients whose procedures were performed laparoscopically. These findings are in line with previous reports and consistent with current guidelines not published when this trial was commended. These patients may be impacted by GDHT, but it should be noted that the incidence of composite complication is impacted by ERPs alone without GDHT. Further research will need to evaluate whether a risk-based algorithm could identify patients who would benefit from GDHT and the associated costs of additional monitoring (Thiele et al., 2016). Accordingly, the design and magnitude of on-going trials would seem justifiable for higher risk patients even in established ERPs (Edwards et al., 2019).

### The present study has both strengths and weakness

A primary weakness is that it was not a prospective, randomized trial, and our data could include sources of bias for which we have not accounted, such as a time bias. We attempted to correct for this, but with an ongoing focus of care standardization and length of stay improvements, we cannot be certain which specific components of care has any causal effect on the outcomes. A strength of this study is the pragmatic, real world implementation of GDHT within an established ERP, as suggested by several consensus guidelines. Yet, this highlights a weakness of our study and possibly of the use of GDHT algorithms in common practice, as we only achieved moderate compliance with the hemodynamic goals. Based on our findings, future trials need to assess whether strict compliance to a GDHT protocol that simultaneously achieves normal preload, afterload, and cardiac output at nearly all timepoints improves outcomes for high-risk patients. If such compliance does improve outcomes, then the means of achieving this level of care in routine practice needs to be determined.

As 80% of postoperative complications are known to occur in 10–15% of patients (Sankar et al., 2015), it is important to define the patients for whom employing additional resources and monitoring is of benefit and whether that benefit can still be realized within an established ERP (Bloomstone & Dull, 2018).

## Conclusions

In a pragmatic pre-post study within an established ERP for colorectal surgery, no benefit was found from implementing GDHT for all patients. Patients with higher co-morbidity and those undergoing open surgery are at higher risk of postoperative complications. Future research should be undertaken in larger trials to assess whether GDHT is of benefit for these patients in addition to general principles of enhanced recovery after surgery.

## Supplementary Information

**Additional file 1: Table S1.** Postoperative American College of Surgeons National Surgical Quality Improvement Program Outcomes. This table lists the postoperative outcomes from the American College of Surgeons National Surgical Quality Improvement Program (ACS NSQIP). **Table S2.** Time in target analysis. This table shows the complete results of time in target analysis.

## Data Availability

The datasets used and/or analyzed during the current study are available from the corresponding author on reasonable request.

## Abbreviations

ACS: American College of Surgeons
ASA: American Society of Anesthesiologists
BMI: Body mass index
CI: Cardiac index
CONSORT: Consolidated standards of reporting trials
CPT: Current procedural terminology
ERP: Enhanced recovery program
GDHT: Goal-directed hemodynamic therapy
IRB: Institutional review board
LOS: Length of stay
MAP: Mean arterial pressure
NSQIP: National Surgical Quality Improvement Program
OR: Odds ratio
PATOS: Present at time of surgery
PDW: Perioperative data warehouse
RM: Ratio of medians
SSI: Surgical site infection
SVR: Systemic vascular resistance
