# Supplementary material for: The effect of adding goal-directed hemodynamic management for elective patients in an established enhanced recovery program for colorectal surgery: results of quasi-experimental pragmatic trial
Source: Perioper Med (Lond). 2020 Nov 23;9:35. doi: 10.1186/s13741-020-00163-3 (PMC7682072; doi:10.1186/s13741-020-00163-3)
Supplement: Supplementary file 1 — Additional file 1: Table S1. Postoperative American College of Surgeons National Surgical Quality Improvement Program Outcomes. This table lists the postoperative outcomes from the American College of Surgeons National Surgical Quality Improvement Program (ACS NSQIP). Table S2. Time in target analysis. This table shows the complete results of time in target analysis. [file 13741_2020_163_MOESM1_ESM.docx]

**Supplemental File**

**Table S1: Percentage of Patients Experiences Postoperative Complications According to the American College of Surgeons National Surgical Quality Improvement Program Definitions**

| **Complication** | **Pre-ERP (N=246)** | **ERP (N=140)** | **ERP+GDHT (N=237)** | **Combined (N=622)** | **P value** |
| --- | --- | --- | --- | --- | --- |
| Surgical Site Infection | 18 (7.3%) | 12 (8.6%) | 11 (4.7%) | 41 (6.6%) | 0.28 |
| Respiratory | 7 (2.8%) | 1 (0.7%) | 3 (1.3%) | 11 (1.8%) | 0.27 |
| Transfusion in 72h | 8 (3.3%) | 3 (2.1%) | 6 (2.5%) | 17 (2.7%) | 0.86 |
| Acute Kidney Injury | 3 (1.2%) | 3 (2.1%) | 0 (0.0%) | 6 (1.0%) | 0.11 |
| Urinary Tract Infection | 2 (0.8%) | 2 (1.4%) | 7 (3.0%) | 11 (1.8%) | 0.21 |
| Sepsis | 8 (3.3%) | 4 (2.9%) | 4( 1.7%) | 16 (2.6%) | 0.54 |
| Cardiac | 2 (0.8%) | 1 (0.7%) | 0 (0.0%) | 3 (0.5%) | 0.45 |
| Deep vein thrombosis | 5 (2.0%) | 2 (1.4%) | 5 (2.1%) | 12 (1.9%) | 0.86 |
| Any Complication | 39 (15.9%) | 19 (13.6%) | 28 (11.9%) | 86 (13.8%) | 0.46 |
| Length of Stay (days) | 4.2 (3.2, 5.9) | 3.4 (3.1, 5.4) | 4.0 (3.1, 5.4) | 4.1 (3.2, 5.4) | 0.10 |
| Readmission within in 7 days of surgery | 11 (4.5%) | 1 (0.7%) | 6 (2.5%) | 18 (2.9%) | 0.09 |
| Readmission within 30 days of surgery | 28 (11.4%) | 19 (13.6%) | 27 (11.4%) | 74 (11.9%) | 0.81 |

Cardiac = stroke, cardiac arrest requiring cardiopulmonary resuscitation, and myocardial infraction; ERP = Enhanced Recovery Program; GDHT = goal-directed hemodynamic therapy; SSI = surgical site infection; Continuous variables are shown in median (25^th^, 75^th^ percentile), categorical data are shown as the number and percentage of patients. P values were calculated using the Wilcoxon rank sum test for continuous variables, and the Kruskal Wallis test for categorical variables. P values for any differences among groups. Definitions come from the American College of Surgeons and can be found here: <https://www.facs.org/-/media/files/quality-programs/nsqip/nsqip_puf_userguide_2018.ashx>.

**Table S2: Time in Target Analysis for Patients Who Received Goal-Directed Hemodynamic Monitoring and Care**

| **Variable** | **No Surgical Site Infection (N = 201)** | **Surgical Site Infection (N = 9)** | **Combined (N = 210)** | **P value** |
| --- | --- | --- | --- | --- |
| Cardiac Index | 86.4% | 76.3% | 85.9% | 0.61 |
| Systemic Vascular Resistance >800 dynes⋅second⋅centimeter ^-5^ | 85.7% | 88.4% | 86.6% | 0.93 |
| Mean Arterial Pressure >65 mmHg | 94.3% | 89.9% | 94.1% | 0.85 |
| Cardiac Index and Systemic Vascular Resistance | 41.1% | 31.9% | 40.6% | 0.60 |
| Cardiac Index and Mean Arterial Pressure | 70.8% | 58.8% | 70.2% | 0.96 |
| Systemic Vascular Resistance and Mean Arterial Pressure | 68.4% | 70.3% | 68.9% | 0.59 |
| 2 out of 3 in target | 95.4% | 94.6% | 94.7% | 0.95 |
| 3 out of 3 in target | 36.1% | 30.5% | 36.1% | 0.54 |

Mean percentage (%) of the time during the intraoperative period that the hemodynamic goals were achieved in isolation and combined at the same time. Goals were cardiac index >2.7 liters/minute/meter body surface area^2^, systemic vascular resistance >800 dynes*seconds*centimeters^-5^, and mean arterial pressure > 65 mmHg. Data are shown in percentage, p values were calculated using the Pearson Chi-square test.
